# Late Cretaceous Aquatic Plant World in Patagonia, Argentina

**DOI:** 10.1371/journal.pone.0104749

**Published:** 2014-08-22

**Authors:** N. Rubén Cúneo, María A. Gandolfo, María C. Zamaloa, Elizabeth Hermsen

**Affiliations:** 1 Museo Paleontológico Egidio Feruglio - CONICET. Trelew, Chubut, Argentina; 2 L. H. Bailey Hortorium, Plant Biology Section, School of Integrative Plant Science, Cornell University, Ithaca, New York, United States of America; 3 Departamento de Ecología, Genética y Evolución, Facultad de Ciencias Exactas y Naturales, Universidad de Buenos Aires, Buenos Aires, Argentina; 4 Department of Environmental and Plant Biology, Ohio University, Athens, Ohio, United States of America; Wesleyan University, United States of America

## Abstract

In this contribution, we describe latest Cretaceous aquatic plant communities from the La Colonia Formation, Patagonia, Argentina, based on their taxonomic components and paleoecological attributes. The La Colonia Formation is a geological unit deposited during a Maastrichtian-Danian transgressive episode of the South Atlantic Ocean. This event resulted in the deposition of a series of fine-grained sediments associated with lagoon systems occurring along irregular coastal plains in northern Patagonia. These deposits preserved a diverse biota, including aquatic and terrestrial plants and animals. The aquatic macrophytes can be broadly divided into two groups: free-floating and rooted, the latter with emergent or floating leaves. Free-floating macrophytes include ferns in Salviniaceae (*Azolla* and *Paleoazolla*) and a monocot (Araceae). Floating microphytes include green algae (*Botryoccocus*, *Pediastrum* and Zygnemataceae). Among the rooted components, marsileaceous water ferns (including *Regnellidium* and an extinct form) and the eudicot angiosperm *Nelumbo* (Nelumbonaceae) are the dominant groups. Terrestrial plants occurring in the vegetation surrounding the lagoons include monocots (palms and Typhaceae), ferns with affinities to Dicksoniaceae, conifers, and dicots. A reconstruction of the aquatic plant paleocommuniy is provided based on the distribution of the fossils along a freshwater horizon within the La Colonia Formation. This contribution constitutes the first reconstruction of a Cretaceous aquatic habitat for southern South America.

## Introduction

There are currently about 2,600 species of vascular aquatic macrophytes distributed among 87 families and 407 genera [1]; these plants inhabit all types of wetland environments and are found throughout the world [2], although they are most diverse in the tropics, a pattern that is particularly pronounced for aquatic angiosperms [3]. Today, vascular aquatic macrophytes are represented by lycophytes (*Isoetes*), ferns (Salviniales or Hydropteridales) [4]–[5], and angiosperms (e.g., Nymphaeales, Nelumbonaceae, and *Ceratophyllum*, to name a few) while non-vascular macrophytes include macroalgae (e.g., *Chara*, *Nitella*), and bryophytes (certain liverworts and mosses) [6]. The fossil record suggests that aquatic ecosystems were conquered by vascular macrophytes in the late Early Cretaceous [7]. Heterosporous water ferns with affinities to the order Salviniales (or Hydropteridales) [4]–[5] first appeared in the latest Jurassic to earliest Cretaceous [8]–[9]–[10]–[11], whereas the earliest aquatic angiosperms, such as *Archaefructus*, *Proteaphyllum*, and ranunculaceous species, appeared in the mid-Early Cretaceous (Barremian-Aptian) [7]–[12]. A greater diversity of aquatic angiosperms, such as Nymphaeales [13]–[14] and some monocots [15]–[16]–[17], appeared later in the Early Cretaceous, when the first unequivocal records of green algae (Hydrodictyaceae, Scenedesmaceae, Coelastraceae and Chlorococcaceae) are also recorded [18]–[19]–[20].

By the Late Cretaceous, freshwater angiosperms were the dominants in aquatic assemblages. For example, aquatic communities documented from Europe [21] include the basal angiosperm *Nymphaeites* (Nymphaeaceae), monocots such as Typhaceae, and the eudicots *Nelumbites* (Nelumbonaceae) and *Palaeoaldrovanda* (Droseraceae), as well as halophytic aquatic plants (although in some cases the affinities of the latter have been questioned) [8]. In Mongolia [22], several Maastrichtian localities of the Nemegt Formation bear aquatic plants that comprise three distinct assemblages: 1- a pondweed assemblage dominated by *Potamogeton*-like plants associated with an isoetalean component; 2- a nymphaealean assemblage; and 3- an assemblage dominated by duckweeds (Araceae subfamily Lemnoideae). These three assemblages occurred in oxbow lake deposits associated with fluvial systems under a temperature regime that was probably subtropical with a monsoonal dry season. Cretaceous and Paleocene aquatic plant communities from western North America and northeastern Asia [23]–[24]–[25]–[26]–[27] resemble modern communities, and include common elements such as *Nelumbites* (Proteales), *Quereuxia* (an angiosperm of unknown affinities), *Cobbania* and *Limnobiophyllum* (monocots in Araceae), along with extinct aquatic ferns (*Hydropteris* in Salviniales) [5].

Comparatively little is known about the evolution of aquatic macrophytes in the Southern Hemisphere, particularly from macrofossil records. Recently, however, a series of contributions describing aquatic macrophytes from the Maastrichtian La Colonia Formation of Patagonia, Argentina, have been published [28]–[29]–[30]–[31]–[32]–[33]. The La Colonia Formation paleoflora, currently documented from three localities that yield macrofossils, is interpreted as preserved *in situ* or after minimal transport given the excellent preservation of delicate plant organs. This interpretation is also supported by the presence of a highly diverse green microalgal component. In this contribution, we summarize recently published and new findings on the composition and environment of the La Colonia Formation paleofloras. This constitutes the first detailed description and ecological interpretation of a latest Cretaceous aquatic plant community from southern South America.

## Materials and Methods

**Figure 1:**
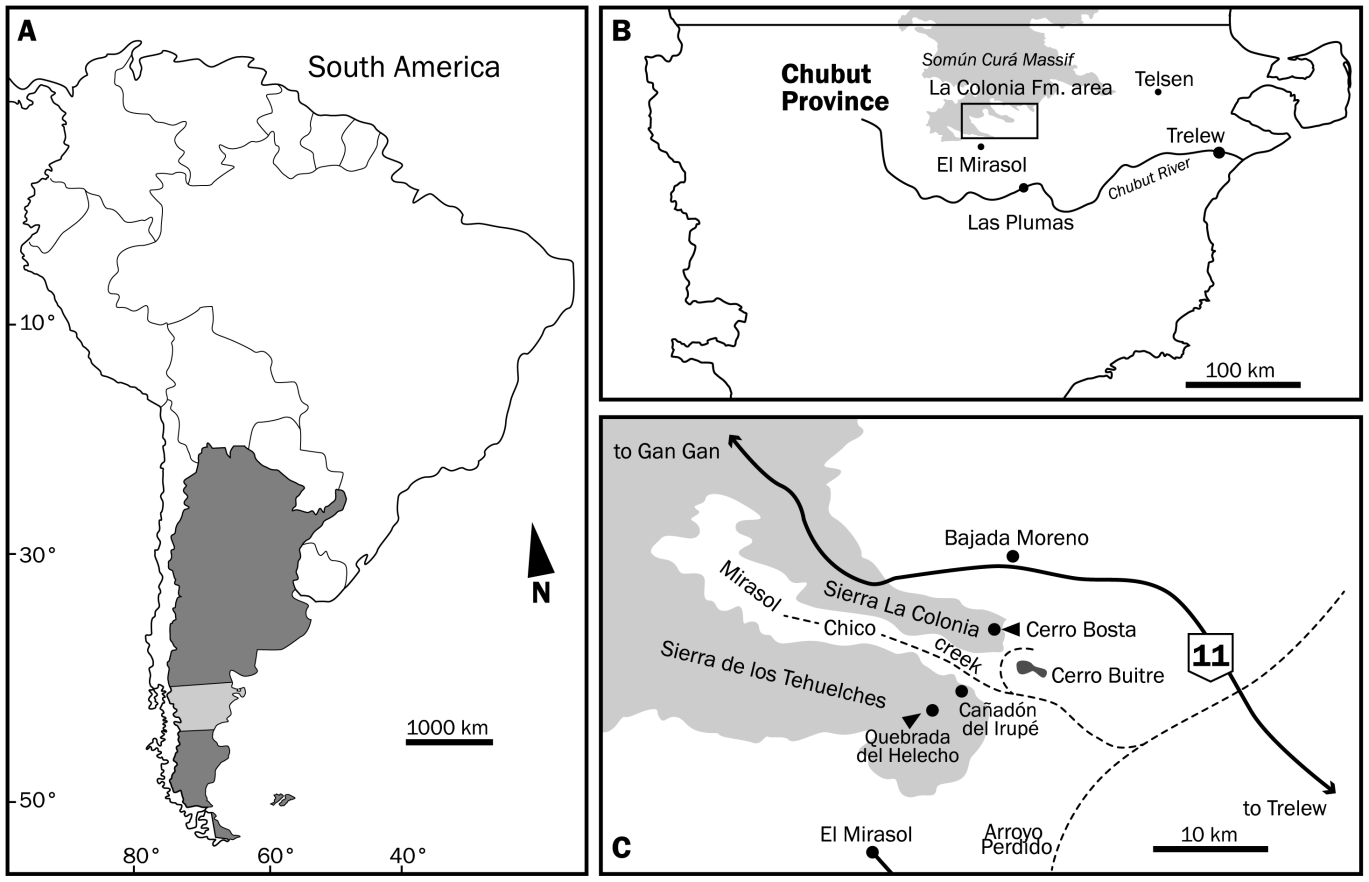
Map showing the location of the outcrops of the La Colonia Formation examined for this study in the Cerro Buitre and Arroyo Mirasol Chico areas. Modified after (101).

**Figure 2:**
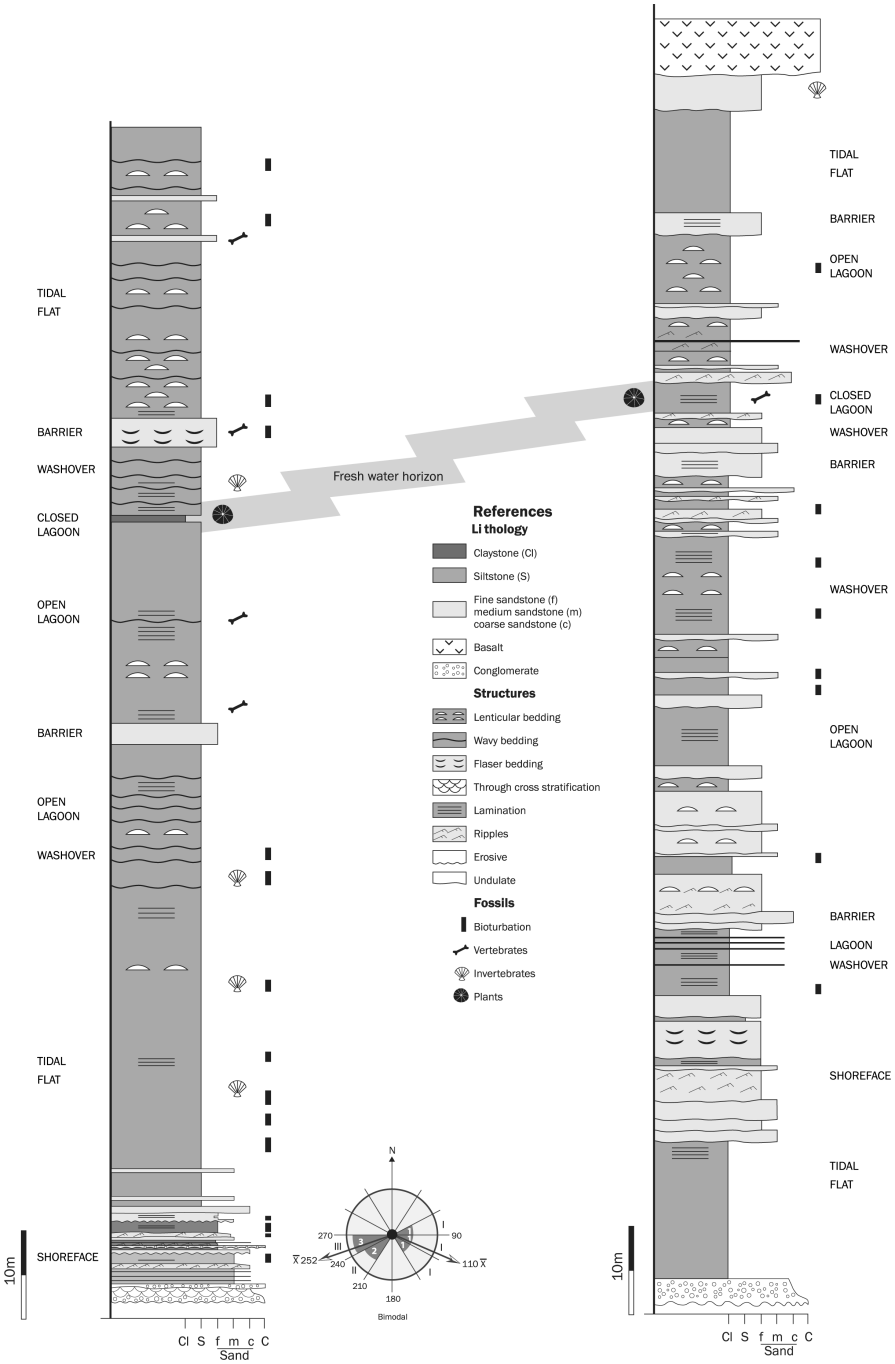
Stratigraphic sections of the La Colonia Formation at the Cerro Bosta locality (a) and Cañadón del Irupé/Quebrada del Helecho localities (b). Note distribution of lagoon facies and fresh water horizon after correlation of aquatic plants.

Macrofossils and palynological samples were collected from several localities of the La Colonia Formation, which outcrops in the central part of northern Chubut Province in Patagonia, Argentina (Fig. 1). Based on the characteristics of its paleobiota, which includes plant macro- and microfossils, dinoflagellates, invertebrates, and vertebrates, the formation is thought to have been deposited during the latest Cretaceous into possibly the earliest Danian [29]–[34]–[35]–[36]. For this contribution, we examined two stratigraphic sections from a plant horizon that can be traced for a distance of approximately 10 km through the La Colonia Formation (Figs. 1, 2). These two sections include localities that yield plant macrofossils: Cerro Bosta and Cañadón del Irupé/Quebrada del Helecho (Fig. 2). The sections were measured from base to top and their sedimentological attributes—including lithologies, sedimentary structures, and fossil content–were noted. Macrofossils and microfossils were identified and their dominance was determined qualitatively. A total of five palynological samples per locality were analyzed from the same horizons as the macrofossils, and they were processed following standard palynological techniques.

No permits were required for the described study. Since the current study is based on specimens previously collected, no particular permit was necessary. This is clearly established in the by-laws of the Chubut Province legislation dealing with fossil protection (Law Number 3559). All macrofossil specimens are housed in the Museo Paleontológico Egidio Feruglio (MEF) Paleobotanical Collection (MPEF-Pb) in Trelew, Chubut, Argentina; palynological samples are also housed in the MEF Palynological collection (MPEF-Mz Palin) and at the Laboratorio de Paleobotánica y Palinología, Facultad de Ciencias Exactas y Naturales, Universidad de Buenos Aires, Argentina (BAFCB). All specimens and data supporting this contribution is freely available throughout public repository as indicated above at www.mef.org.ar/index.php?option = com_content&view = article&id0102&ltemid085&lang = es.

### Geology and sedimentology of the La Colonia Formation

#### Background

The La Colonia Formation is a sedimentary accumulation that outcrops at the southeastern edge of the Somuncurá Massif in northern Patagonia, Argentina [37] (Fig. 1). The Somuncurá Massif (or Plateau) is a structural unit composed of Paleozoic metamorphic and igneous complexes, which are the source rocks for clastic Late Cretaceous/early Paleogene sedimentary deposits occurring in the region. A major transgression caused by subsidence of the south Atlantic margin took place during the Late Cretaceous, which gave rise to extensive shallow epicontinental seas defined as Patagonian Platforms [35]–[38]. These seas covered most of the Patagonian region and represented the last episode of a tectonic foreland stage [39]. Thus, a series of Late Cretaceous coastal plain sedimentary deposits representing estuaries, deltas, and lagoon/barrier complexes accumulated [36]–[40].

Deposits of the La Colonia Formation have a maximum thickness of approximately 240 m [37]. They are underlain by late Early to Late Cretaceous sediments of the Chubut Group or the Puntudo Chico Formation [41], and are exposed in the Cerro Buitre/Mirasol Chico Creek area (Fig. 1), and towards the NE in the Telsen area over the volcanics of the Jurassic Marifil Formation [36]. This Jurassic unit seems to have played an important role in shaping the topography of the landscape during the latest Cretaceous transgression [36], probably as part of the Atlantic Patagonian Dorsal [38]. The La Colonia Formation is overlain by basalts of the El Buitre Formation (Paleocene) and the informally named “Baibian Beds” (probably Eocene to early Oligocene) to the southwest [42]. Above these is a basaltic intrusion (Somuncurá Formation), which is succeeded by the classic Oligocene-early Miocene deposits of the Sarmiento Formation. The whole sequence in this area is crowned by Neogene basalts and Quaternary sedimentary deposits (Tehuelches Pebbles).

The sedimentology of the La Colonia Formation has been characterized based on data collected from several localities. Three lithofacies associations were initially described along the eastern slope of the La Colonia Formation in the proximity of the Cerro Buitre area (Fig. 1) [34]. The lowest association is composed of coarse-grained sandy to conglomeratic cross-bedded deposits up to 16 m thick and was initially interpreted as a terrestrial setting drained by low- to moderately sinuous fluvial channels [34]. The middle and thickest association is represented by fine-grained deposits and includes three facies (laminated mudstones, pelite-fine sandstones, and banded siltstones and claystones) thought to have been deposited on coastal plains. The upper association is a single facies consisting of laminated claystones deposited in an intertidal zone.Two of the three facies (pelite-fine sandstones and banded siltstones and claystones) have also been recognized within the middle facies association at a locality north of Cerro Buitre (Fig. 1), where the La Colonia Formation is almost 130 m in thickness [43]. Near Telsen (Fig. 1), the La Colonia Formation is much thinner (around 15 m on average) and starts with fine conglomerates that represent an erosive surface (or ravinement surface) [36]–[44]–[45] resulting from the Maastrichtian transgression. This is overlain by three sandstone facies and two fine-grained facies that define four depositional environments: 1) shoreface transitioning to off-shore, 2) tidal flats, 3) lagoons with tidal influence, and 4) lagoons without marine influence (36). The presence or absence of marine influence was determined by the presence or absence of dinoflagellates.

#### New sedimentological results

Although the sedimentology of the La Colonia Formation has received detailed examination and interpretation, the major sedimentological processes responsible for the deposition of the La Colonia Formation are still poorly understood. The presence of an aquatic paleobiota in the formation is thus significant, as it is helpful for making paleoenvironmental interpretations. In this regard, we have analyzed two plant-bearing sections that correspond to the Cerro Bosta and Cañadón del Irupé/Quebrada del Helecho localities of the La Colonia Formation (Fig. 1). These sections have quite similar characteristics in terms of their thickness and in the facies associations represented. Sandy/conglomeratic coarse-grained deposits that represent the initial transgressive phase of the latest Cretaceous Atlantic Epicontinental Sea occur at the base of each section, and correlate to the erosive “key surface” (or ravinement) represented in localities to the northeast [36]. These deposits are 5 to 10 m thick and are interpreted as representing shoreface sedimentation dominated by bi-modal wave processes (Fig. 2a). This is contrary to the initial fluvial interpretation [34], but in agreement with that suggested from study of sediments in the Telsen area [36]. Notably, embayed transgressing coastlines normally lack fluvial influence in their barrier-lagoon systems [46]–[47].

The La Colonia Formation section measured at the Cañadón del Irupé/Quebrada del Helecho localities (Fig. 2b) is 137 m thick. It represents barrier island deposits that prograde over lagoonal deposits, creating water bodies separated from the nearshore. The barrier deposits are composed of fine- to medium-grained, laminated to cross-bedded sandstones. In some cases, laminated coarse silts to very fine sandstone deposits represent sandbars. The lagoon deposit proper is represented by fine-grained laminated to massive sediments (clays and silts), in some cases with high participation of heterolithic facies (lenticular and linsen bedding). These deposits, due to lack of terrestrial sediment supply, are associated with marine influence, in particular by washover caused by stormwaters entering the lagoon [48]. Washover and lagoonal environments tend to be preserved during transgressions [49], when organic muds are vertically accreted in a “shore zone” (Fig. 2a) [50]–[51]. The absence of washover channels or tidal inlets that represent connections between the open sea and the lagoons is probably due to the presence of a microtidal regime that resulted from shallow conditions along the shoreline of the Patagonian Platform sea [38]. Microtidal barrier islands tend to be long and narrow, with abundant storm wave-dominated washover deposits (Fig. 2b) that overtop the barrier [52]. This communication with the open sea is responsible for salinity changes within the lagoons, with development of aquatic plant communities during freshwater stages [53]. Therefore, coastal environments show a normal gradation from saline to freshwater conditions [54].

The section at the Cerro Bosta locality reaches 135 m in thickness (Fig. 2a) and has a well-developed shoreface facies that clearly shows a bi-modal paleocurrent pattern, suggesting tidal and wave influence. Here, barrier/washover deposits are not as evident, and most of the section appears to be dominated by the constant influence of tidal/wave processes. The exception is the horizon that yields the aquatic fossil plants, the presence of which suggests freshwater conditions. As noted for the Cañadón del Irupé/Quebrada del Helecho section, the absence of both coarse-clastic sediments and sedimentary structures indicates that the site was relatively distant from active fluvial dynamics.

In summary, the La Colonia Formation outcropping between the Cerro Bosta locality and the Cañadón del Irupé/Quebrada del Helecho, includes sedimentary deposits interpreted as clastic coastal plains bathed by shallow seas. Barrier-island/lagoon complexes developed in this region. The lagoons were protected from the sea by barrier islands, and were built by wave-dominated beach/shoreface processes normally related to low sediment supply and sea-level rise [55].

### Reconstruction of the aquatic plant community of the La Colonia Fm

Modern aquatic macrophytes rarely occur as monospecific populations in freshwater systems, but instead tend to form recognizable assemblages composed of several species representing several different major life forms. These forms include free-floating macrophytes [2], also known as pleustophytes [4]–[56], that are not anchored to the substrate and float in the water column or on the water surface; and rooted macrophytes [2] that are anchored to the substrate with structures like rhizomes or roots and known as benthophytes [56] or, perhaps more commonly, rhizophytes [4]. Rhizomatous or rooted macrophytes can further be divided into those with leaves that are completely submerged, those with emergent leaves (borne above the surface of the water), and those with leaves floating on the water surface [2]–[57]. The La Colonia Formation plant community clearly includes both free-floating macrophytes as well as rooted/rhizomatous macrophytes with either emergent or floating leaves. Additionally, there is a diversity of microphytes in the form of free-floating green algae (Chlorophyta).

#### Free-floating microphyte component

**Figure 3:**
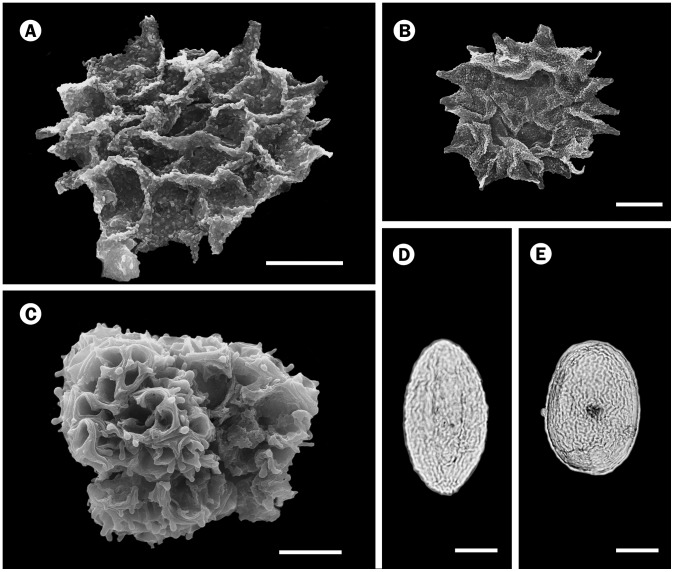
Free-floating microphyte components (chloroccocalean microalgae). A, B (SEM): *Pediastrum* spp.; BAFCB meb 262. C: *Botryoccocus* sp. (SEM); BAFCB meb 262. D, E: Zygnemataceae spores (LM); BAFCB pm 260. Scales A, B, C = 1000 µm; D, E = 10 µm.

Green microalgae (Fig. 3), represented by members of the families Hydrodictyaceae (*Pediastrum* spp.) (Fig. 3 A,B), Botryococcaceae (*Botryoccocus* spp.) (Fig. 3C), and filamentous Zygnemataceae (*Spirogyra* type, *Zygnema* type, and *Mougeotia* type) (Fig. 3 D, E), comprise the microphyte component of the plant community. These planktonic algae are abundant and diverse in sediments of the localities studied, suggesting highly favorable conditions for their growth, which likely occurred in humid to wet substrates during long hydroperiods. Abundant freshwater algae indicate meso- to eutrophic conditions, strong light penetration, and small- to medium-sized bodies of water [58]. No dinoflagellate cysts were found within the microfossil samples, suggesting a lack of marine influences at these levels.

Botryococcaceae colonies are today widely dispersed in temperate and tropical regions and are able to tolerate seasonally cold climates [58]. They normally live in freshwater (although sometimes in brackish water), occurring in bogs, temporary pools, ponds, and lakes. Quiet water environments are favorable to their proliferation, and they may form a thick scum on the water surface [58].

The fossil record in South America indicates that from the Late Cretaceous to the Early Eocene only the widespread *Pediastrum boryanum* (Hydrodictyaceae) occurs [59]. In this regard, the presence of at least three species in the La Colonia sediments denotes that the genus was well established by the late Cretaceous at high southern latitudes. *Pediastrum* is a freshwater planktonic coenobium that can occur in various trophic conditions [59], although theyare generally more abundant under slightly eutrophic conditions. Proliferation of *Pediastrum* may result in the formation of an ooze at the bottom of a lagoon, pond, or lake. Cenobia walls have a delicate sporopollenin layer that is easily broken during diagenetic processes. Nevertheless, most cenobia found in the La Colonia sediments remain complete and well preserved, denoting *in situ* preservation in a low energy paleoenvironment.

Zygnemataceae, one of the most common families of modern freshwater unbranched filamentous algae, typically occurs in shallow, stagnant, clean, and oxygen-rich waters where they form scums on the water surface, although a few species occur in brackish water. Members of this family produce sexual spores (zygospores) in spring under favorable conditions and also asexual resistant spores (aplanospores) that allow them to persist through periods when environmental conditions are unfavorable. Differentiation between sexual and asexual spores is not discernible in most genera but their sole presence indicates local conditions of the body of water [60]–[61].

#### Free-floating macrophytes

The free-floating macrophyte [2] or pleustophyte [6]–[56] component of the La Colonia plant paleocommunity is represented by vascular plants, incudingferns and angiosperms (see Table 1). The vascular plants can be classified as acropleustophytes, or macrophytes that float on the water surface [6]–[56], based on their structure and comparison to related modern plants.

**Table 1:** Distribution and abundances of plant taxa represented by micro- and macrofossils at the three localities studied.

Fossil taxa recorded	Botanical Affinity	Habit-Source	Cerro Bosta			Cañadón del Irupé			Quebrada del Helecho		
			Fossil type			Fossil type			Fossil type		
			m	ms	M	m	ms	M	m	ms	M
**Pteridophyta**											
*Azolla*-type	Salviniaceae	fl-au	++			+++	+		+		
*Crybelosporites* cf. *C. pannuceus* (Brenner)	Marsileaceae	fl-au	+			+					
*Cyathidites australis* Couper	Dicks/Cyath/Schiz	terr-all	+++			++			+		
*Dicksonia*-type	Dicksoniaceae	terr-all						++			++
*Gleicheniidites senonicus* Ross	Gleicheniaceae	terr-all	+			+			+		
*Lugiomarsiglia aquatica* Gandolfo et al.	Marsileaceae	fl-au						+			
*Mirasolita irupensis* Hermsen et al.	Marsileaceae	fl-au						+			+
*Molaspora lobata* (Dijkstra) Hall	Marsileaceae	fl-au		+			+				
*Paleoazolla patagonica* Archangelsky et al.	Salviniaceae	fl-au	+	+					+		
*Regnellidium thomas-taylorii* Cúneo et al.	Marsileaceae	fl-au			+						
**Gymnosperms**											
*Classopollis* sp. 1	Cheirolepidiaceae	terr-all	++			+++					
*Classopollis* sp.2	Cheirolepidiaceae	terr-all	++			+++			+		
Leaves and twigs	af. Cheirolepidiaceae	terr-all						+			
*Lygistepollenites* sp.	Podocarpaceae	terr-all	+			+					
*Microchacrydites* sp.	Podocarpaceae	terr-all	+			+					
*Podocarpidites* spp.	Podocarpaceae	terr-all	+			+			+		
**Angiosperms**											
*Arecipites* spp.	Arecaceae	terr-all	+			+			+		
*Inaperturotetradites* sp.	Monocotyledoneae	fl-au	+			+					
*Intratriporopollenites* sp.	Malvaceae	terr-all	+			+					
Leaf remains	Araceae	fl-au						+			
Leaf remains	Arecaceae	terr-all						+			
*Nelumbo puertae*	Nelumbonaceae	fl-au						+			
*Pandaniidites* sp.	Araceae	terr-all				+					
*Peninsulapollis* sp.	Proteaceae	terr-all	+			+					
Reproductive structures	Nelumbonaceae	fl-au						+			
*Sparganiaceaepollenites* sp.	Typhaceae	fl-au	+			+					
*Typha*-type leaves	Monocotyledoneae	fl-au						+			
Other dicots	Several families	terr-all	+			+		+	+		
**Algae**											
*Botryococcus braunii*	Botryococcaceae	fl-au	+++			++			+		
*Botryococcus* sp. 1	Botryococcaceae	fl-au	+++			++					
*Pediastrum boryanum*	Hydrodictyaceae	fl-au	+++			++					
*Mougeotia*-type	Zygnemataceae	fl-au	+								
*Spirogira*-type 1	Zygnemataceae	fl-au	+								
*Spirogira*-type 2	Zygnemataceae	fl-au	+			+					
*Zygnema*-type	Zygnemataceae	fl-au	+								
*Pediastrum* sp. 1	Hydrodictyaceae	fl-au	+++			++			+		
*Pediastrum* sp. 2	Hydrodictyaceae	fl-au	+++			++					
**Fungi**											
Spores		terr-all	++			+++					

Main micro and macroplant fossil record from La Colonia Formation. m = microfossil, ms = mesofossil, M = macrofossil. Abundances: + = present, ++ = common, +++ = abundant. Habit-taphonomy: fl-au = floating-autochthonous, terr-all = terrestrial-allochthonous.

**Figure 4:**
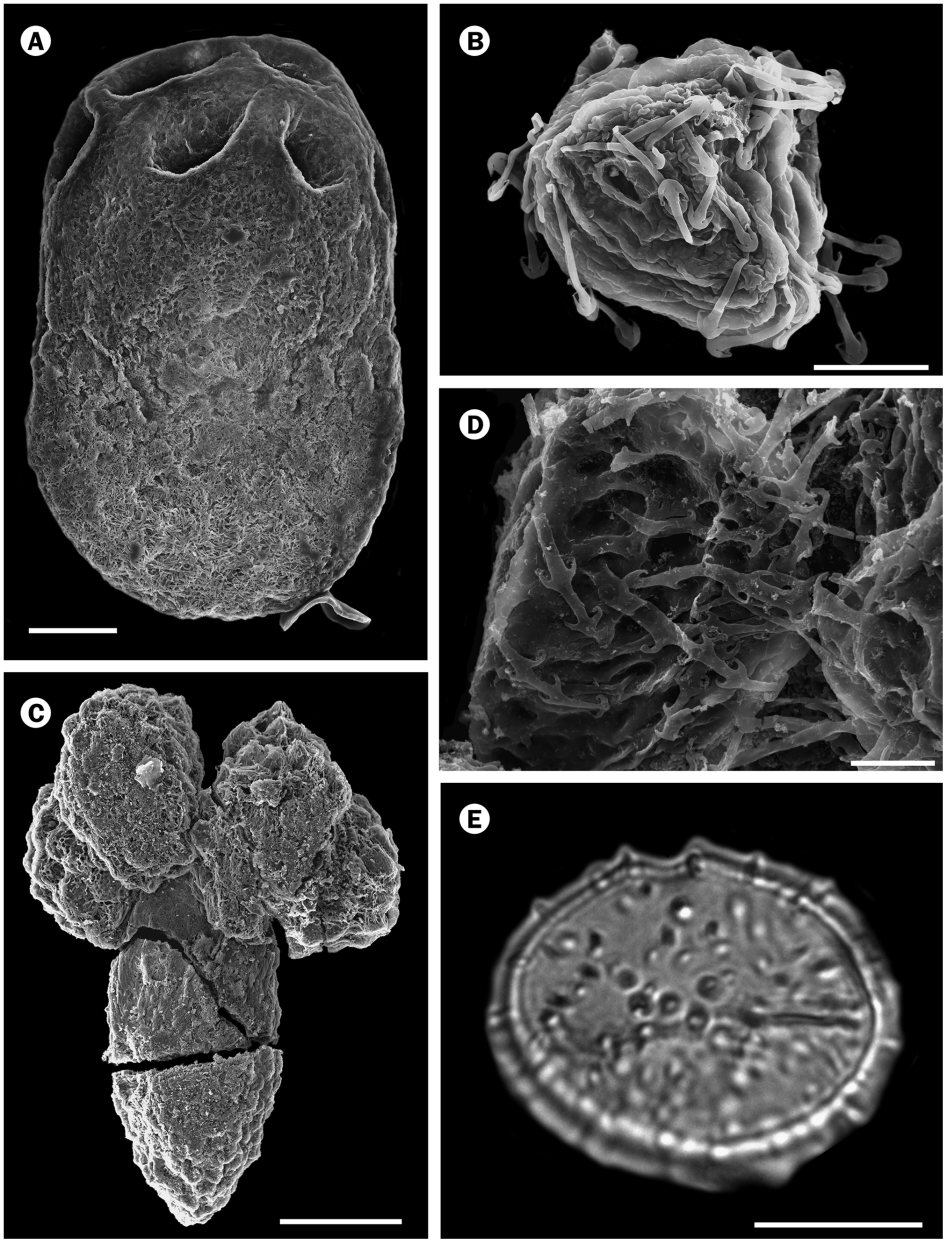
Spores and pollen organs representing free-floating aquatic macrophytes. A: *Azolla*-like megaspore (SEM) without floats preserved; MPEF-MEB 5505 (scale = 50 µm). B: *Azolla* microspore massula (SEM); BAFCB meb 263 (scale = 20 µm). C: *Paleoazolla patagonica* megaspore with floats preserved (SEM); MPEF-MEB 5506 (scale = 200 µm). D: *Paleoazolla patagonica* microspore massula (SEM); MPEF-MEB 5507 (scale = 10 µm). E: Araceae pollen grain (*Pandaniidites* sp.); BAFCB pm 259 (LM) (scale = 10 µm).

**Figure 5:**
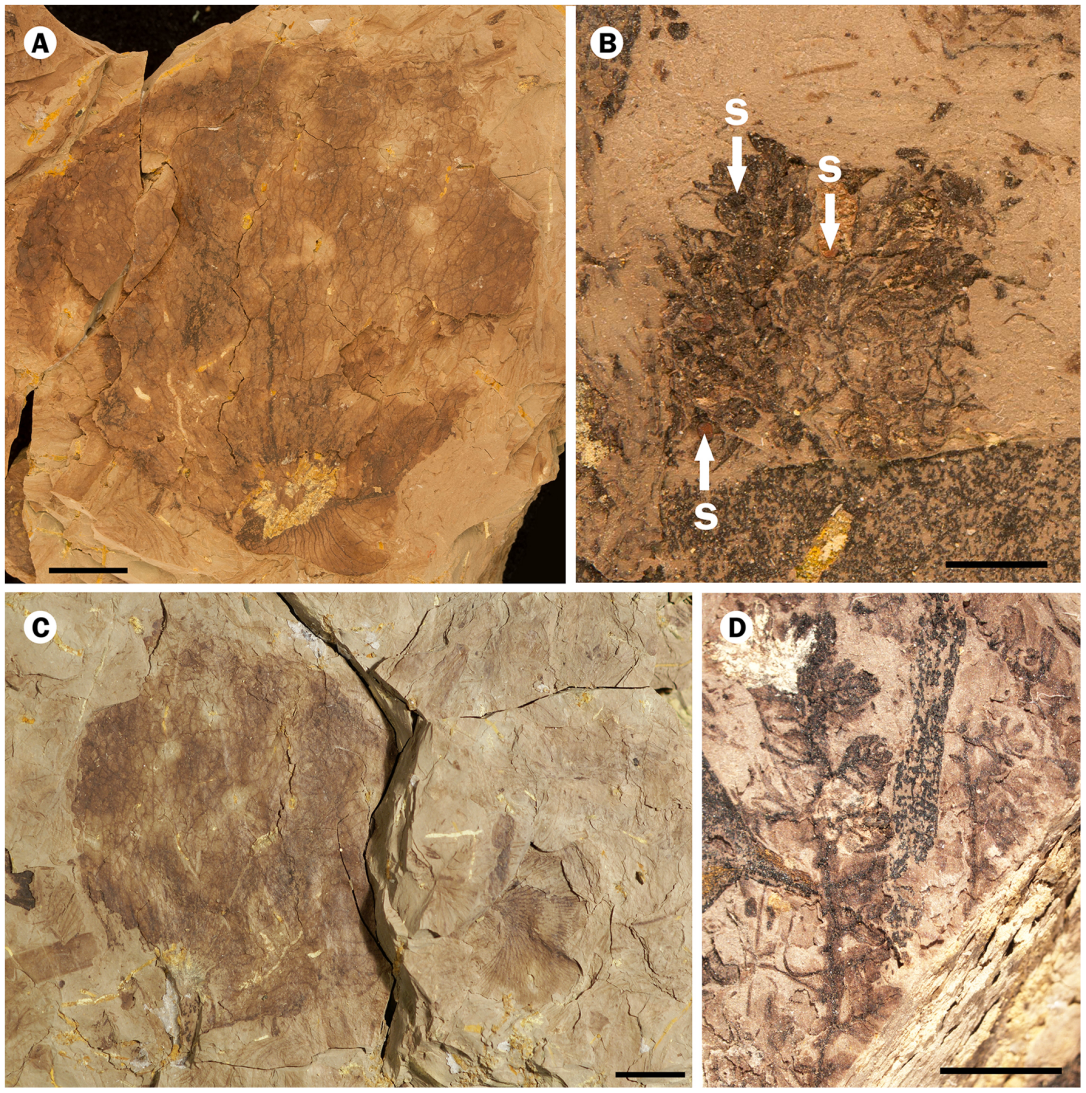
Free-floating aquatic macrophyte components from the Cañadón del Irupé locality. A, C: Lemnoid leaves; MPEF-Pb 3983. B, D: *Azolla* sporophytes B. Sporophyte with sporocarps (s, arrows); MPEF-Pb 5060. D: Sterile sporophytes; MPEF-Pb 5081. Scales A, C = 1 cm; B, D = 5 mm.

The ferns are members of the family Salviniaceae (Order Salviniales, water ferns). Modern and fossil Salviniaceae are aquatic, free-floating, heterosporous ferns [62]–[63]. Salviniaceous ferns were first identified from the La Colonia Formation based on megaspores and microspore massulae assigned to *Paleoazolla patagonica* from the Cerro Bosta locality [28]. *Paleoazolla patagonica* megaspores with preserved floats and microspore massulae with characteristic glochidia bearing recurved lateral barbs were found at all three La Colonia Formation localities sampled for this study (Figs. 4 C, D). *Azolla* megaspores without floats preserved and microspore massulae bearing glochidia with anchor-shaped ends like those found in modern *Azolla* section *Azolla* [64]–[65] were also found at the Cerro Bosta and Cañadón del Irupé localities (Fig. 4 B). Additionally, *Azolla*-like sporophytes, including sterile and fertile material, are known from the Cañadón del Irupé locality (Figs. 5B, D) [30]. The fertile specimens bear megasporocarps and microsporocarps, the latter including smaller round bodies interpreted as *in situ* microspore massulae (Fig. 4 B). The spores to which the sporophyte correspond have not yet been definitively established.

Today, *Azolla* is frequently found in abundance floating on the surface of ponds, lakes, marshes, and slow-moving streams, in freshwater or sometimes in brackish water. It occurs in tropical to warm-temperate regions throughout the world [62]–[66] and has limited tolerance for frost, salinity, and drought [67]–[68]–[69]. Due the fact that *Azolla* does not tolerate high salinity and prefers stagnant water, it is an excellent indicator of freshwater, low energy environments [62]–[63]–[69].

Another free-floating component of this plant community is represented by leaves and pollen attributed to a lemnoid Araceae basal to the duckweed clade and definitely part of the free-floating aquatic aroids [31] (Figs. 5 A, C). The leaves belong to a newly described genus comparable to the fossil genera *Limnobiophyllum* and *Cobbania* [30]–[31]. They are associated with *Pandaniidites*-type pollen (Fig. 4E), which has also been found *in situ* in *Limnobiophyllum* anthers [70]. These fossils are found only at the Cañadón del Irupé locality [31]. Today, the Araceae are a large, mostly terrestrial tropical family with approximately 20 species adapted to the aquatic/wetland environment with low flow or stagnant shallow waters [56]–[71] as interpreted for the Quebrada del Irupé locality in Patagonia.

#### Rooted/rhizomatous macrophytes

The rooted macrophyte [1] or benthophyte or rhizophyte [4]–[57] component of the La Colonia Formation flora is also represented by ferns and angiosperms (Table 1). These plants are thought to have had either emergent or floating leaves based on their structure and comparison to their nearest living relatives.

**Figure 6:**
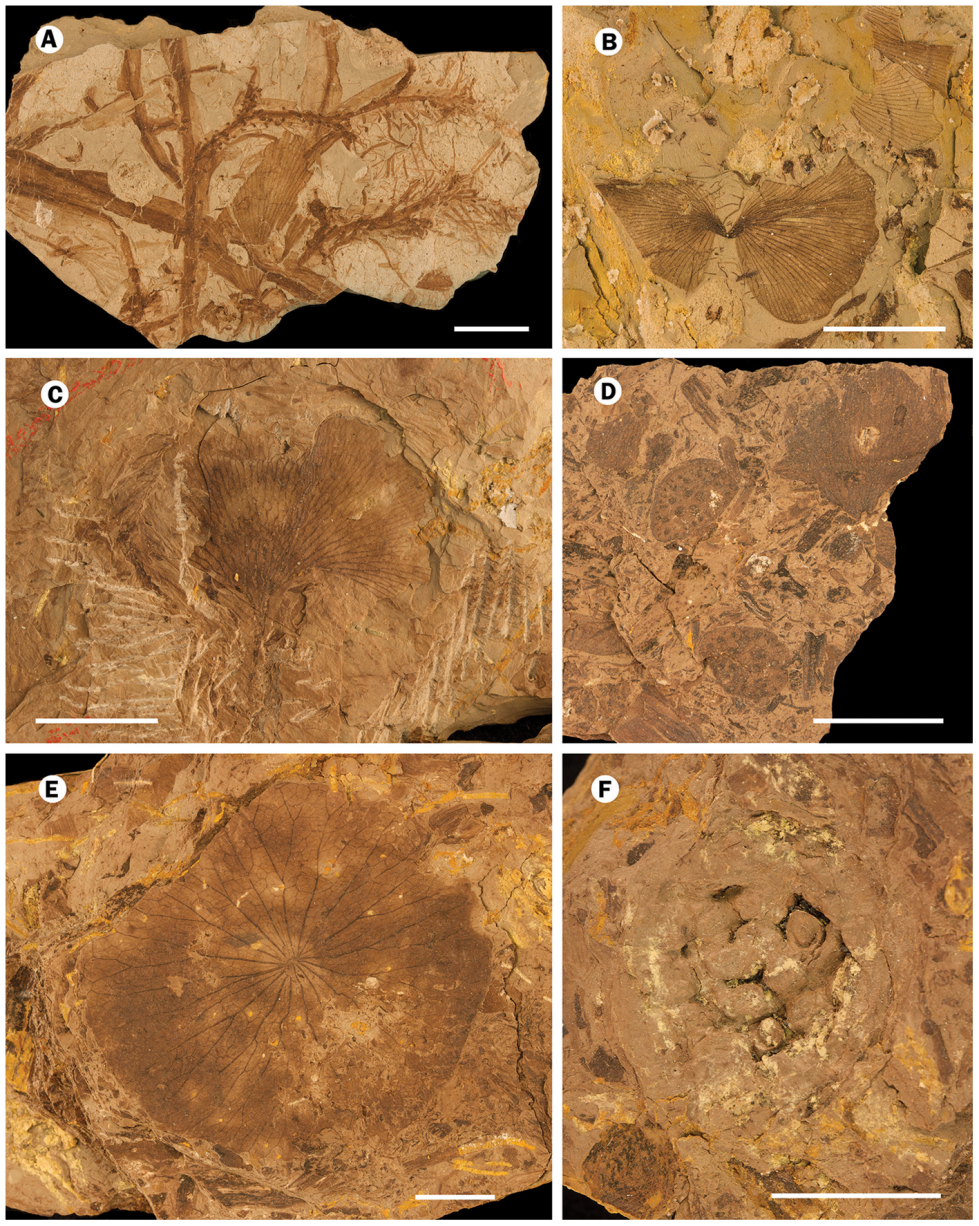
Rooted/rhizomatous macrophytes. A: *Regnellidium thomas-taylorii* rhizomes, rootlets, petioles and leaflets; MPEF-Pb 5405. B: *R. thomas-taylorii* leaflets; MPEF-Pb 5446. C: *Mirasolita* leaflets; MPEF-Pb 5521. D: *Luigimarsiglia* sporocarps; MPEF-Pb 5527. E: *Nelumbo puertai* leaf; MPEF-Pb 864. F: *Nelumbo*-like associated reproductive organ; MPEF-Pb 920. Scales = 1 cm.

**Figure 7:**
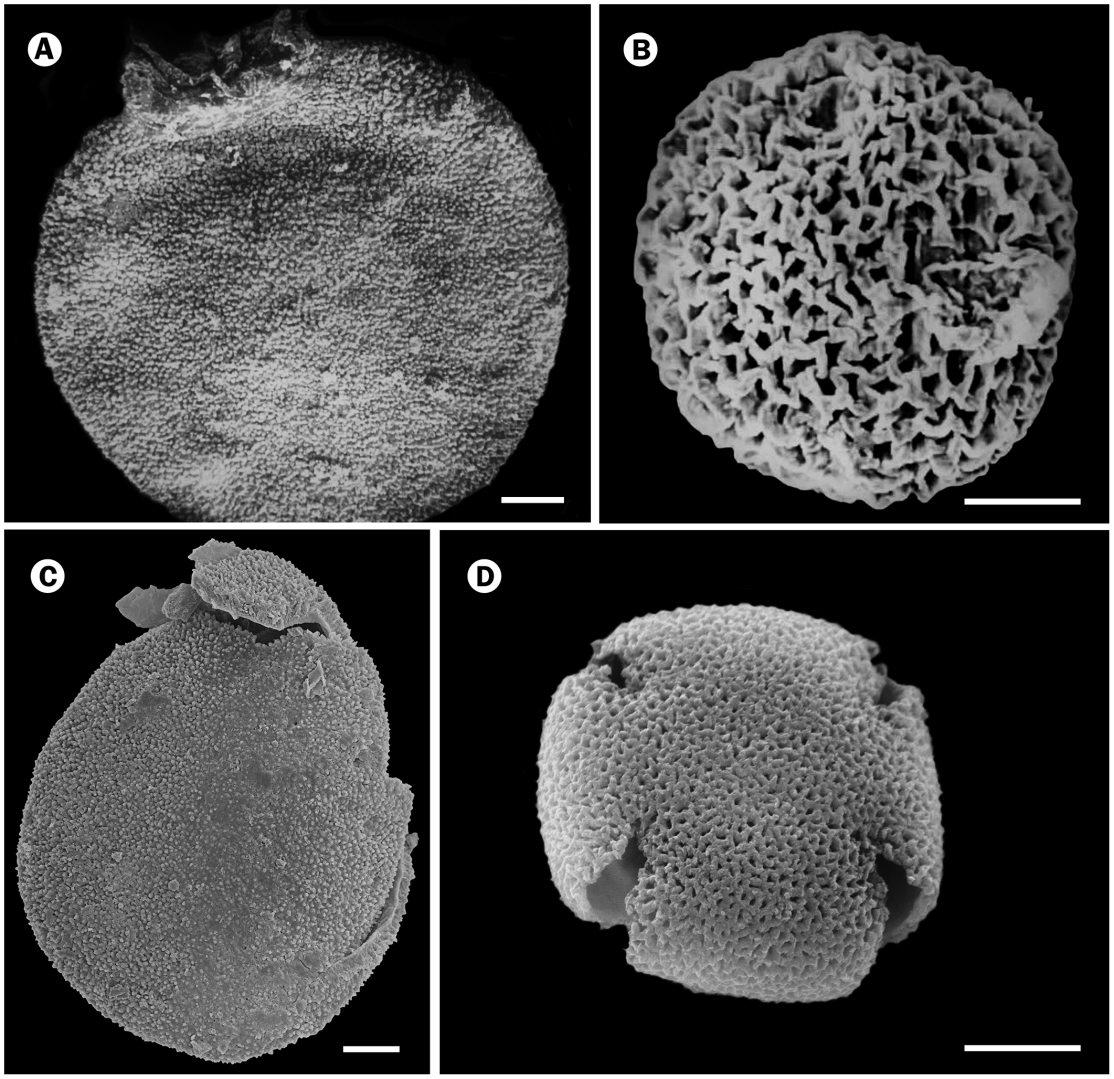
Rooted/rhizomatous macrophytes represent by spores and pollen. A: *Molaspora lobata* megaspore (SEM) associated with *Regnellidium thomas-taylorii*; MPEF-MEB 5501 (scale = 50 µm). B: *Crybelosporites* microspore (SEM) associated with *Regnellidium thomas-taylorii*; MPEF-MEB 5502 (scale = 10 µm). D: *Molaspora lobata* megaspore (SEM) associated with *Mirasolita* and *Luigimarsiglia*; MPEF-MEB 5503 (scale = 50 µm). D: *Nelumbo*-like tetracolpate-reticulate pollen grain (SEM); BAFCB pm 263 (scale = 10 µm).

Ferns include members of the family Marsileaceae (Order Salviniales, water ferns). The fossil species *Regnellidium thomas-taylorii* (Fig. 6 A, B) is the only macrophyte known from two of the localities, Cerro Bosta and Quebrada del Helecho. Remains of this plant include petiolate compound leaves with two leaflets (Fig. 6B), aerenchymatous rhizomes bearing rootlets (Fig. 6 A), and a small septate sporocarp. The marsileaceous *sporae dispersae Molaspora lobata* (megaspores, Fig. 7A) and *Crybelosporites* (microspores, Fig. 7B) were recovered in abundance from the same layer bearing *R. thomas-taylorii* macrofossils. *Mirasolita irupensis*, leaves and leaflets representing an extinct form (Fig. 6C, D), co-occur with *Lugiomarsiglia aquatica*, representing marsileaceous sporocarps (Fig. 6D), at the Cañadón del Irupé locality; *Molaspora lobata* megaspores are known to occur in the same layers [33].

Living marsileaceaous ferns are amphibious and rhizomatous, with adventitious roots borne on the rhizome that anchors them in the substrate [72]–[73]. All three extant genera of Marsileaceae (*Marsilea*, *Pilularia*, and *Regnellidium*) are adapted to intermittently dry conditions [74]–[75]. Extant *Marsilea* and *Regnellidium* exhibit heterophylly, producing two to three adult leaf forms (terrestrial, floating, and submerged) depending on the availability of water when the leaves develop [76]. These leaves vary in some of their features in characteristic ways. For example, floating leaves tend to be glabrous and entire-margined, whereas terrestrial leaves tend to bear trichomes and may have terminal teeth [77]–[78]. The morphology of the fossil leaves suggests that they are floating or terrestrial/emergent forms. *Regnellidium* only grows in shallow waters or in muddy substrates that are subject to periodic flooding and the leaf petioles reach a maximum of ca. 21 cm in length [74], roughly indicating the maximum water depth in which the extant plants might occur when the leaves develop.

Sporocarps of *Marsilea* and *Regnellidium* typically develop on the leaf petioles only when the plants are exposed to air by a decrease in water level, and it is thought that sporocarp production may be stimulated by exposure of plant organs to light. Water, however, is necessary for dehiscence [76]. The leaflets of *Marsilea* leaves that bear sporocarps die away by the time the sporocarps are mature [76], so perhaps the *Mirasolita* leaves and *Lugiomarsiglia* sporocarps found at the Cañadón del Irupé locality were produced at different times under slightly different environmental conditions. The somewhat inexplicable paucity of marsileaceous sporocarps in the fossil record, despite the occurrence of marsileaceous ferns in habitats conducive to preservation and the durability of the sporocarps themselves, is perhaps explained by the tendency of sporocarps to float or to germinate quickly under favorable conditions [78].

The eudicot *Nelumbo puertae* is another macrophyte of the La Colonia Formation flora that has emergent or floating leaves. This taxon occurs at the Cañadón del Irupé locality [29]–[30]. The plant is represented by typical peltate, discoidal leaves (Fig. 6E) and associated infructescences (Fig. 6F). Modern Nelumbonaceae comprise two species within the genus *Nelumbo*. Modern *Nelumbo* develops horizontal rhizomes with nodal adventitious roots and tubers. Its leaves are emergent or floating, with petioles up to 2 m long. Flowers are also elevated and frequently emergent on a peduncle up to 2 m in length. The maximum lengths of the leaf petioles and flower peduncles determine the maximum depths at which this aquatic plant can grow [54]–[79]. Considering the size and mature condition of the nelumbonaceous remains from Cañadón del Irupé, the lagoon system at this locality likely could not have been deeper than 2 m.

#### Structure and organization of the aquatic paleocommunity

Aquatic macrophyte assemblages are commonly organized along depth gradients, with macrophytes bearing emergent leaves typically dominating shallow areas, submerged macrophytes colonizing deeper sites, androoted or rhizomatous species with floating leaves inhabiting zones of intermediate depth [80]. Other physical environmental variables can also affect the distribution and community structure of aquatic plants, the most relevant being water temperature light availability, and salinity [53]–[81]. Most aquatic macrophytes and some microalgae are able to tolerate conditions unfavorable for their growth and survival (e.g., suboptimal water temperatures, changes in water chemistry, low water levels, etc.) for limited periods.

**Figure 8:**
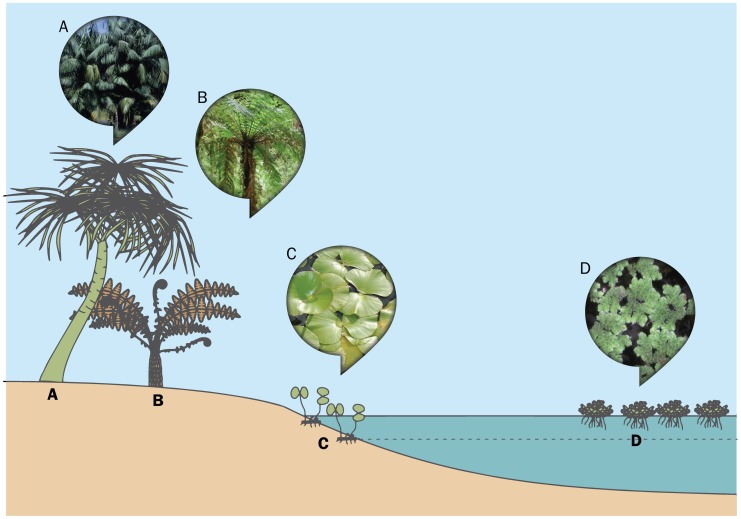
Reconstruction of the plant communities at the Cerro Bosta locality. Surrounding (close-allochthonous) terrestrial vegetation: (a) Arecaceae, (A) *Euterpe* (Plantsystematics.org- DOL11633) and (b) Dicksoniaceae, (B) *Dicksonia sellowiana* (Photo by R. Moran, Plantsystematics.org- DOL75000). Autochthonous vegetation: Emergent-rooted macrophyte (c) *Regnellidium thomas*-*taylorii*, (C) *Regnellidium diphyllum* (Photo by D.W. Stevenson, Plantsystematics.org- DOL25635) and free-floating macrophyte (d) *Azolla* and *Paleoazolla*, (D) *Azolla* (Photo by R. Moran, Plantsystematics.org- DOL6803).

**Figure 9:**
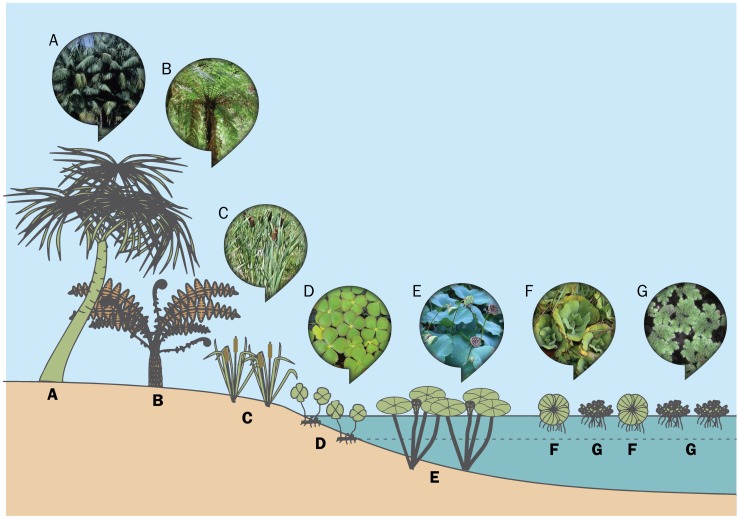
Reconstruction of the plant communities at Cañadón del Irupé/Quebrada del Helecho localities. Surrounding (close-allochthonous) terrestrial vegetation: (a) Arecaceae, (A) *Euterpe* (Plantsystematics.org- DOL11633), (b) Dicksoniaceae, (B) *Dicksonia sellowiana* (Photo by R. Moran, Plantsystematics.org-DOL75000), and (c) *Typha*-like, (C) *Typha latifolia* (Photo by K.C. Nixon, Plantsystematics.org- DOL6212). Autochthonous vegetation: Emergent-rooted macrophytes: (d) *Mirasolita*-*Lugiomarsiglia*, (D) *Marsilea scalaripes* (Photo by D. Nickrent, PhytoImages.siu.edu-DOL69348) and (e) *Nelumbo puertae* and reproductive structures, (E) *Nelumbo lutea* (Photo by R. Moran, Plantsystematics.org- DOL42110); and freeifloating macrophytes: (f) Araceae, (F) *Pistia lactucoides* (Photo by K.C. Nixon, Plantsystematics.org- DOL4884) and (g) *Azolla* and *Paleoazolla*, (G) *Azolla* (Photo by R. Moran, Plantsystematics.org- DOL6803).

The aquatic plant communities at the Cerro Bosta and Quebrada del Helecho localities were dominated by the rooted marsileaceous fern *Regnellidium thomas-taylorii*, which was associated with the presumably free-floating salviniaceous fern *Paleoazolla* and abundant microalgae (see reconstructions in Figs. 8, 9). These localities probably represented a sector of a lagoon system with eutrophic conditions and shallow water. At the Cañadón del Irupé locality (see reconstruction in Fig. 9), aquatic macrophytes were more diverse, with free-floating components represented by an araceous plant and water ferns in the family Salviniaceae (*Azolla* and *Paleoazolla*). There were also free-floating microphytes and rooted macrophytes, the latter including marsileaceous ferns and the eudicot *Nelumbo* (Nelumbonaceae). Based on the life habits of the nearest living relatives of these plants, this paleocommunity also probably occupied shallow waters of the lagoon system. The substratum may have been more organic than at the other localities as indicated by the carbonaceous nature of the fossil remains. The aquatic plant horizon identified in different sections of the La Colonia Formation suggests that freshwater conditions prevailed at this stage throughout the whole region. Periodic influx of saline water from the nearby sea caused retreat of freshwater macro- and microphytes to more suitable habitats in the coastal plain water bodies. Finally, the lack of frost tolerance in modern analogues of some components of the La Colonia Formation flora (e.g., *Azolla*, lemnoids, Marsiliaceae) suggests that water temperatures were rarely, if ever, too cold.

#### Surrounding vegetation

Terrestrial vegetation surrounding the lagoon system can be divided into two prinicipal types based on the participation of different plant groups, the “wetlands” (or parautochthonous elements) and the “nearby upper lowlands” (allochthonous elements).

#### 1.Wetlands or parautochtonous (marshes)

**Figure 10:**
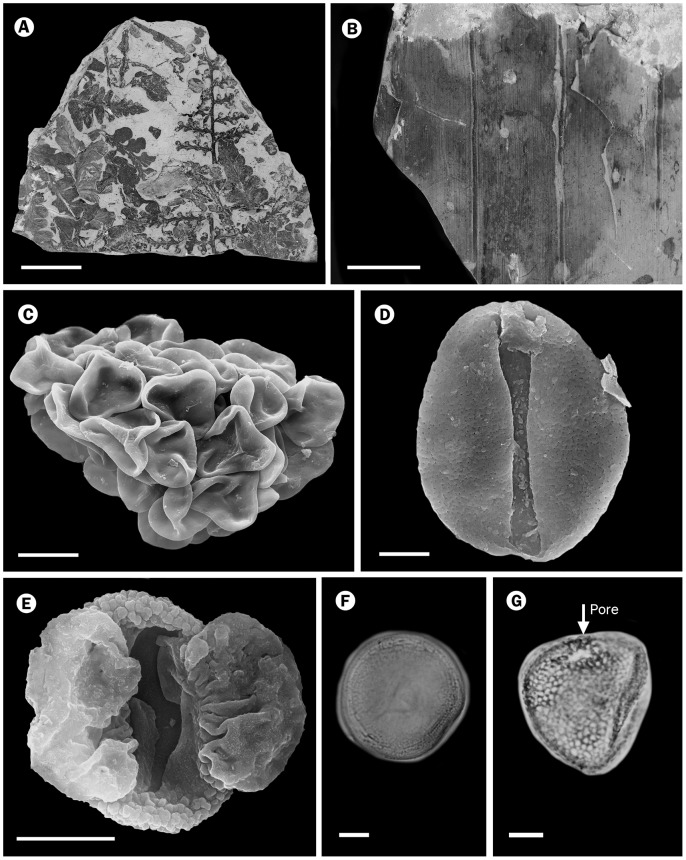
Main representatives of the surrounding lowland vegetation. A: Dicksoniaceous fertile and sterile fronds; MPEF-Pb 6457. C: Dicksoneaceous spore massulae; BAFCB meb 263. B: Typhaceous leaf; MPEF-Pb 5074. D: *Arecipites* type pollen grain; BAFCB meb 261. E: Podocarpaceous bisaccate pollen grain; BAFCB meb 262. F: *Classopolis* pollen grain; BAFCB meb 259. G: Typhaceous pollen grain; BAFCB meb 260. C, D, E (SEM); F, G (LM). Scales: A, B = 1 cm; C = 20 µm; D = 5 µm; E, F, G = 10 µm.

This terrestrial component is represented in the microflora by arecaceous (palm) and typhaceous pollen and in the macroflora by herbaceous monocots (probably Typhaceae) and several dicot leaves of uncertain affinities [30] (Table 1; Fig. 10). Dicksoniaceous ferns, represented by macro- and microfossils (Fig. 10A, C), are an important terrestrial component of the vegetation. We interpret these plants as having occupied a belt of vegetation surrounding the water bodies where a shallow water table was present. They probably represent a region of open vegetation composed of an understory of dicot shrubs or small trees (represented by leaf macrofossils) and herbaceous monocots (Typhaceae), a tree-fern stratum (Dicksoniaceae), and an overstory of taller palm trees (Arecaceae) (Fig. 8).

Extant genera of Typhaceae (*Typha* and *Sparganium*) are aquatic macrophytes; they can be considered caulescent planemergent or emergent benthophytes [57] or submerged/floating-leaved/emergent macrophytes [2]–[82]–[83]–[84]–[85]. A few fragments of parallel-veined, putatively typhaceous leaves were reported from the Cañadón del Irupé locality [30] (Fig. 10B). However, the presence of typhaceous plants is mostly based on several pollen grains of *Sparganiaceaepollenites*, a type similar to pollen produced by *Sparganium* and *Typha* [86], found at the Cerro Bosta and Cañadón del Irupé localities (Fig. 10D). Typically, Typhaceae grow in freshwater lagoons and stagnant ponds, although they sometimes occur in brackish environments or in swamps. Modern Typhaceae produce enormous amounts of pollen, and pollen grains that land in the water eventually sink and settle on the substrate [87]–[88]. This suggests that *Spargiaceaepollenites* pollen should be abundant if plants were producing it *in loco* at the La Colonia Formation localities. Because *Sparganiaceaepollenites* pollen was found in very small amounts within the palynoflora, we infer that the typhaceous plants were not growing within the lagoon itself, but were part of the vegetation belt surrounding it.

Macrofossil Dicksoniaceae are extremely abundant at the Quebrada del Helecho locality and occur more sporadically at the Cañadón del Irupé locality [30]. At both localities, dicksoniaceous ferns are represented by dispersed spores and spore massulae as well as fertile and sterile tripinnate pinnules (Fig. 10A). While the sporophytic phase is completely terrestrial in Dicksoniaceae, these ferns favor humid environments and are frequently found on riverbanks [89]–[90].

Palm (Arecaceae) pollen grains were found in sediments of the study localities as isolated grains (Fig. 10G) as well as clumps. When palm pollen is found in clumps, it is indicative that the pollen grains fell close to the source [91]. Although few species of palms grow in wetlands, several are characteristic of this type of environment. These include *Manicaria saccifera*, *Mauritia flexuosa*, and *Euterpe oleracea*, which today are quite common in the Orinoco Delta region of Venezuela [92], where they occur in coastal freshwater swamps, frequently in large, dense stands [92].

#### 2. Nearby upper lowlands (allochtonous)

The plant remains of the nearby upper lowland flora are considered to be allochthonous. They are composed of plants that did not require direct contact with a permanent water body and were adapted to a deeper water table. The upper lowlands were inhabited primarily by conifers, in particular those yielding *Classopollis* (Cheirolepidiaceae) and podocarpaceous pollen (Fig. 10F). Some angiosperms were also present, as suggested by their dispersed pollen grains and undetermined leaf remains. Pollen include three species of Nothofagaceae, several types of Proteaceae, and several other unidentified eudicots and monocots.

### Plant consumers: the associated fauna and its environmental and paleoecological implications

Undoubtedly, one of the best-preserved and most diverse latest Cretaceous meso- and macrofaunas in the Southern Hemisphere occurs in the La Colonia Formation. The fauna is composed of dinosaurs including carnosaurs, hadrosaurs, and ankylosaurs [93]–[94], aquatic and terrestrial turtles [95], plesiosaurs [96]–[97], snakes [98], birds [99], and mammals [34]–[100]–[101]. The presence of polycotylid plesiosaurs [96]–[97] and elasmosaurs [96] in the Cerro Bosta paleofauna supports the interpretation of lagoonal settings for these sections of the formation. Polycotylid plesiosaurs [102] are part of a derived clade of small plesiosaurs (actually pliosaurs) that lived in lagoonal, estuarine and freshwater environments [103]. Elasmosaurs are another group of small plesiosaurs that lived in the same types of paleoenvironments.

Of particular interest among the dinosaurs are several forms with aquatic or semi-aquatic lifestyles, the hadrosaurs and ankylosaurs. Hadrosaurs are traditionally considered important consumers of aquatic plants based on their ecology and adaptations, while ankylosaurs could have been consumers of aquatic plants as suggested from their dentition features adapted mainly for soft vegetation [104]. Turtles are the most abundant and diverse of the reptilian fauna in the La Colonia Formation, and some of them are also potential consumers of the aquatic vegetation. Plants such as green microalgae colonies, *Azolla* (a fast-growing fern that is also a good source of nitrogen due to its symbiosis with a cyanobacterium), and aquatic angiosperms could have served as nutritious sources of food for these herbaceous vertebrates [65].

True water bugs (Heteroptera, Nepomorpha) are also commonly found in the La Colonia Formation (Petrulevicius, pers. comm.). Nepomorphs are distributed worldwide, but are most diverse in the tropics, inhabiting different habitats ranging from shores of small ponds and banks of streams to deep inland water bodies [105]. These insects are characterized by their seasonal adaptations that permit them to survive under periodically fluctuating environmental conditions such as those that affect lagoon systems, especially during episodes when the lagoon is flooded with seawater [105]. In the La Colonia Formation, these water bugs most likely found shelter and food sources in the aquatic vegetation, probably creating their own microhabitats.

### Biogeographic patterns

Most diverse modern aquatic plant communities are primarily distributed in the zone between the 30°N and 30°S latitude [1]. Species diversity is highest in the Neotropics, followed by the Afrotropics, Indo-Malaya, the Nearctic, and then the remaining phytogeographic regions [1]. The broad distribution of aquatic plants has classically been attributed to long-distance dispersal, in particular via migratory birds [2]. Continental drift and land connections also likely played a major role, and were probably particularly important before the evolution of birds in the Cretaceous [24].

This tendency toward broad geographic distribution was established early in the history of aquatic macrophytes. Fossil Nelumbonaceae, for example, are mainly recorded from Laurassia with a curious record in southern South America at the La Colonia and Lefipán formations by the Late Cretaceous [29]–[30], initially explained by the spreading of the group [106]–[107], but suggesting continuous and similar conditions in fresh water coastal ecosystems from North to South America that would have allowed some migratory flow. Interestingly, the record of the family in South America is only represented by fossil forms from Patagonia [29]–[30], with the current presence of *Nelumbo* on Gondwanan continents (northern South America and northern Australia) probably being attributable to human-mediated dispersal [107]. Additionally, aquatic marsileaceous ferns are found throughout the world in the Cretaceous, with an extensive and cosmopolitan dispersed spore record that extends throughout the period (for summaries of many known occurrences, see *Arcellites*, *Molaspora*, *Rodeites* and *Crybelosporites* in [9]–[108]). Aquatic Araceae are clearly present by the latest Cretaceous to Paleocene not only in South America, but also in North America and northeastern Asia [23]–[27]–[70]–[109]–[110]

Latest Cretaceous and Paleocene aquatic communities reported from the Northern Hemisphere include those from Europe (19), Mongolia/NE Asia [22]–[24], and western North America [24]–[27], which share elements with the La Colonia flora. Although the taxonomic composition of these floras is not the same at generic level, commonalities are observed at the level of plant family or plant habit. All are composed of floating microalgae (e.g., *Pediastrum*), floating aquatic ferns (e.g., *Azolla*), rooted salvinialean ferns (e.g., *Hydropteris*, Marsileaceae), and floating and/or rooted angiosperms (Araceae and Nelumbonaceae), with a terrestrial component consisting of selected coniferous gymnosperms, ferns, and angiosperms. The presence of aquatic swamp communities dominated by *Azolla* and *Pediastrum* was reported from the latest Cretaceous Hell Creek Formation [24], North Dakota, USA, in paleoenvironments analogous to modern subtropical swamps. These elements are abundant in the Cañadón del Irupé/Quebrada del Helecho localities, suggesting a similar environment.

In Patagonia, an aquatic plant community has been detected from the nearbyPaleocene (Danian)-aged Bororó Formation [111]. This community comprises some of the elements found within the La Colonia flora, including Chlorococcales, Zygnematales, Salviniaceae, pteridophytes, and conifers, as well as angiosperms in the families Araceae, Arecaceae, Typhaceae, and Proteaceae. The occurrence of the same taxa suggests that the Cretaceous/Paleogene boundary event did not affect aquatic plant communities, which retained approximately similar structure and composition during the transition between the Maastrichtian and the basalmost Paleocene (Danian). Similar conclusions were obtained from the coeval and nearby Lefipán Formation in northwestern Patagonia [112].

### Concluding remarks

Near the end of the Cretaceous in central and southern Patagonia, marine transgressions favored oceanic climates that influenced the composition of the vegetation. In general, the Patagonian paleofloras were dominated by angiosperms, suggesting warm-temperate to subtropical humid conditions for the region [113]. Notably, the frost intolerance of the modern analogues of some plants found in the La Colonia Formation sediments—such as Salviniaceae, Marsiliaceae, Araceae, and palms—also indicates a tropical to subtropical climate.

Based on the data presented in this report, it is clear that by the latest Cretaceous there was a suite of lagoonal coastal wetlands in Patagonia, that promoted the proliferation and, later, the preservation of freshwater plant communities. The interpretation of the paleofloral localities as representing a lagoonal environment is confirmed by the presence and types of free-floating aquatic macrophytes (pleustophytes), free-floating microphytes, and rooted macrophytes with floating or emergent leaves (planmergent or emergent benthophytes) that constituted the main components of the aquatic vegetation. A small number of terrestrial and aquatic macrophytes surrounded the water bodies, and additional allochthonous components occurred on higher ground. The clastic conditions of these southern hemisphere mid-latitude environments did not allow for the development of mangrove vegetation that is normally linked to more calcareous shallow platforms.

Many of the records reported for the La Colonia paleocommunity are of significant value. The Salviniaceae, Nelumbonaceae, and Araceae constitute the first macrofossil records for these Southern Hemisphere families. The occurrence of arecaceaous pollen within a wetland environment suggests that palms were found in both drier and in wetland areas in Patagonia in the past. In summary, the La Colonia paleocommunity is the first aquatic plant community described for the Southern Hemisphere based on both macro- and microfossils, and, thus, provides a unique window into better understanding the Late Cretaceous Patagonia and Southern Hemisphere.
